# Models Based on the Mitscherlich Equation for Describing Typical and Atypical Gas Production Profiles Obtained from In Vitro Digestibility Studies Using Equine Faecal *Inoculum*

**DOI:** 10.3390/ani10020308

**Published:** 2020-02-17

**Authors:** Christopher D. Powell, Mewa S. Dhanoa, Anna Garber, Jo-Anne M. D. Murray, Secundino López, Jennifer L. Ellis, James France

**Affiliations:** 1 Department of Animal Biosciences, University of Guelph, Guelph, ON N1G 2W1, Canada; 2 Institute of Grassland and Environmental Research, Plas Gogerddan, Aberystwyth SY23 3EB, UK; 3 College of Medical, Veterinary and Life Sciences, School of Veterinary Medicine, University of Glasgow, Glasgow G61 1QH, UK; 4 Departamento de Producción Animal, Universidad de León, E-24007 León, Spain; 5 Instituto de Ganadería de Montaña, CSIC-Universidad de León, Finca Marzanas s/n, 24346 Grulleros, Spain

**Keywords:** gas production technique, in vitro digestibility, Mitscherlich equation, feedstuff evaluation, fermentation kinetics, substrate degradation

## Abstract

**Simple Summary:**

Feedstuff evaluation through animal trials is time consuming and expensive. An alternative, the gas production method, measures the amount of fermentation gas produced from incubating feedstuffs with microbes from ruminal fluid or faecal samples. Models can be applied to gas production profiles to determine extent of feedstuff degradation either in the rumen or in the hindgut. Typical gas production profiles show a monotonically increasing monophasic pattern. However, atypical gas production profiles exist whereby at least two consecutive phases of gas production are present; these profiles are much less well described. Two models are proposed to fit these biphasic profiles, a sum of two Mitscherlich equations, and sum of Mitscherlich + linear equations. Additionally, two models that describe typical monophasic gas production curves, the simple Mitscherlich and the generalised Mitscherlich (root-*t*) model, were assessed for comparison. Models were fitted to 25 gas production profiles resulting from incubating feedstuffs with faecal *inocula* from equines. Of these 25 profiles, 17 displayed atypical biphasic patterns, and 8 displayed typical monophasic patterns. The two biphasic models were found to describe both the atypical and typical gas production profiles accurately. These models allow for the evaluation of feedstuffs using cost- and time-efficient methods.

**Abstract:**

Two models are proposed to describe atypical biphasic gas production profiles obtained from in vitro digestibility studies. The models are extensions of the standard Mitscherlich equation, comprising either two Mitscherlich terms or one Mitscherlich and one linear term. Two models that describe typical monophasic gas production curves, the standard Mitscherlich and the France model [a generalised Mitscherlich (root-*t*) equation], were assessed for comparison. Models were fitted to 25 gas production profiles resulting from incubating feedstuffs with faecal *inocula* from equines. Seventeen profiles displayed atypical biphasic patterns while the other eight displayed typical monophasic patterns. Models were evaluated using statistical measures of goodness-of-fit and by analysis of residuals. Good agreement was found between observed atypical profiles values and fitted values obtained with the two biphasic models, and both can revert to a simple Mitscherlich allowing them to describe typical monophasic profiles. The models contain kinetic fermentation parameters that can be used in conjunction with substrate degradability information and digesta passage rate to calculate extent of substrate degradation in the rumen or hindgut. Thus, models link the in vitro gas production technique to nutrient supply in the animal by providing information relating to digestion and nutritive value of feedstuffs.

## 1. Introduction

The in vitro gas production technique [1,2] is widely applied in animal nutrition for ranking and evaluating feedstuffs. This technique is based upon the assumption that the gas produced from incubating a feedstuff with a microbial *inoculum* is the consequence of the anaerobic fermentation of that feedstuff [3]. In ruminant nutrition, gas production profiles generated have been used in conjunction with the retention time of digesta (derived from the rate of passage) to determine extent of degradation in the rumen [3,4,5,6,7,8,9]. In equine nutrition, the technique has been proposed as an in vitro surrogate for determining the digestibility and nutritive value of feedstuffs using in vivo methods [10,11,12,13,14].

Typical gas production profiles are diminishing returns or sigmoidal in shape (see [5] for illustration), and France et al. [4] derived a purpose-built function in the form of a generalised Mitscherlich equation with an additional root-*t* term to represent a variable fractional rate of degradation for fitting to a wide range of curve shapes. This model is commonly referred to as the “France” model, and this term will be used herein. However, atypical patterns have also been recorded. Groot et al. [15] reported biphasic profiles and selected a function comprising two generalised rectangular hyperbolae to fit them, while other atypical patterns have been observed by research workers though not formally reported in the scientific literature. Interpretation of these atypical patterns include the autonomous fermentation of feed components in incubated feedstuffs, with these feed components representing chemical or nutritional fractions, with total gas produced being a summation of gas produced from each fermented feed component [16].

The Mitscherlich equation has a long history of application in the agricultural sciences and in applied biology generally, both as a response function and as a growth function [17,18]. The Mitscherlich, which is an expression of the principle of the Law of Diminishing Increments as originally applied to the effect of fertilization on crop yields, is a function that reaches an asymptotic maximum and represents diminishing returns behaviour in rising to the asymptote. It is a special case of the function proposed by France et al. [4]. In this paper, we consider four types of gas production profile (diminishing returns, sigmoidal, biphasic and asymptotic, biphasic but non-asymptotic). The profiles considered were obtained from incubating feedstuffs with faecal *inocula* from equines using the gas production method of Theodorou et al. [2]. These data were taken from two experiments with either grazing horses or ponies fed primarily grass hay. The main objective of this paper was to assess the ability of the simple Mitscherlich, and three extensions of this classical function, to describe both typical and atypical gas production profiles. The functions were derived to describe gas production profiles on the basis of substrate degradation, rather than on the basis of gas produced, permitting the estimation of fermentation kinetic parameters. Using relatively simple equations, proposed herein, extent of feedstuff degradation in the hindgut of equines can be calculated using model parameter estimates in conjunction with information regarding substrate degradability and digesta passage rate. Therefore, a secondary objective was to compare how model fits, and by extension model derived parameters, affect extent of feedstuff degradation values when these models are applied to mono- and bi-phasic gas production profiles.

## 2. Materials and Methods

### 2.1. Datasets

#### 2.1.1. Experiment 1: Inoculum from Horses

In a study to assess the fermentative capacity of faecal *inocula*, Murray et al. [19] sourced *inoculum* from 14 grass-kept horses (maintained on grass 24 h a day) from the International League for the Protection of Horses in Norfolk, UK. *Inocula* were prepared from these 14 horses—7 of them predisposed to laminitis and the other 7 clinically normal—so that the effect of laminitis on hindgut fermentative activity could be evaluated. Grass hay was the substrate incubated in vitro. Due to the large distance to the laboratory, *inoculum* was stored at −20 °C for transportation on ice. *Inocula* were subsequently thawed and incubated at 38 °C. Gas production was recorded using the method of Theodorou et al. [2] and three replicates per *inoculum* were used. Standard in vitro gas production results were described by Murray et al. [19]. The grass hay data yielded 14 gas production profiles, one for each horse, as the average over the three replicates. Visual inspection of these profiles revealed a predominance of atypical patterns.

#### 2.1.2. Experiment 2: Inoculum from Ponies

The study comprised a total of eleven different *inocula* (see Table 1 for details). Garber et al. [20] sourced eight *inocula* from Welsh Section A geldings arranged in a 4 × 4 Latin square experimental design aiming to investigate the in vitro fermentation of high fibre/high concentrate diets supplemented with yeast (control diets with no yeast). Another 3 faecal *inocula* were obtained in an experiment in which ponies were fed a grass hay only diet (control), or the same grass hay supplemented with increasing concentrations of a fibrolytic enzyme (either 0.75 or 3.75 mL of enzyme solution per kg DM hay). Gas production was recorded using the ANKOM RF gas production system [21] and three replicates per *inoculum* were used. Preliminary results were reported by Garber et al. [20]. The data yielded 11 gas production profiles, one for each treatment, after averaging the three replicates for each *inoculum*. Visual inspection revealed both typical and atypical patterns.

The entirety of the observed gas production values (Dataset 1–25) used in this study can be found in the Supplementary Information section of this paper, Table S1.

### 2.2. Models Fitted

The classical Mitscherlich equation used in crop science has the general form:

$$y=A-\left(A-B\right)e^{-ct}$$

where the ordinate *y* is crop yield, the abscissa *t* is fertilizer rate, and *A*, *B* and *c* are constants. The parameter *A* represents the asymptotic value of *y* (i.e., maximum yield) and *B* the minimum yield (i.e., no fertilizer application).

For application to the gas production technique, the ordinate becomes cumulative gas production (mL) and the abscissa becomes time since inoculation (h). Cumulative gas production at zero time can be considered negligible and a lag *T* ≥ 0 (h) may occur before onset of fermentation, so the Mitscherlich equation becomes:

(1) $$y=A\left(1-e^{-c\left(t-T\right)}\right);t\geq T$$

In this equation, *A* would represent the asymptotic gas production (mL) and *c* (h^−1^) the fractional rate of fermentation. In this paper, we explore Equation (1) and three different extensions (Equations (2)–(4) below) of this classical function for use in describing typical and atypical gas production profiles.

Gas production profiles are typically monophasic, asymptotic and often sigmoidal (e.g., [5]). France et al. [4] derived the following equation from rate:state principles to describe such profiles:

(2) $$y=A\left\{1-\exp\left[-c\left(t-T\right)-d\left(\sqrt{t}-\sqrt{T}\right)\right]\right\};t\geq T$$

Here, *A* (mL) is the asymptotic value of *y*, and *c* (h^−1^) and *d* (h^−0.5^) are fractional rate constants. Equation (1) is a special case of Equation (2) (i.e., *d* = 0). This equation is commonly referred to as the France model.

Biphasic, asymptotic gas production profiles have also been observed (e.g., [15]), and these would appear to lend themselves to description by the sum of two Mitscherlich terms:

(3) $$y=A_{1}\left(1-e^{-c_{1}\left(t-T_{1}\right)}\right)+A_{2}\left(1-e^{-c_{2}\left(t-T_{2}\right)}\right);t\geq T_{1},\;t\geq T_{2}$$

The first term in Equation (3) is zero until time *T*_1_ and likewise the second term until time *T*_2_. Equation (1) is also a special case encompassed by Equation (3) (i.e., *A*_2_ = 0). This latter equation will be referred to as the double Mitscherlich model.

As mentioned above, instances of profiles that do not exhibit typical asymptotic behaviour have also been observed but not formally reported. Such profile forms suggest a function resulting from the sum of a Mitscherlich term and a linear term might provide an appropriate description:

(4) $$y=A\left(1-e^{-c\left(t-T_{1}\right)}\right)+\beta \left(t-T_{2}\right);t\geq T_{1},\;t\geq T_{2}$$

where the parameter *β* (mL h^−1^) is the slope of an underlying linear trend. As for Equation (3), the first term in Equation (4) is zero until time *T*_1_ and likewise the second term until time *T*_2_. Putting *β* = 0 in Equation (4) yields Equation (1). This latter equation will be referred to as the Mitscherlich + linear model.

### 2.3. Extent of Degradation

The extent of degradation (*E*) of substrate in a specific compartment or region of the gastro-intestinal tract may be calculated from the gas production curve, provided the *inoculum* used to generate the profile is representative of that compartment. If the profile is diminishing returns in shape, first-order kinetics with a constant fractional rate of degradation describes substrate degradation and Equation (1) can be fitted to the profile. Extent of degradation is then given by:

(5) $$E=S_{0}e^{-kT}c/\left[\left(c+k\right)\left(S_{0}+U_{0}\right)\right]$$

where *S*_0_ (g) is the amount of the incubated substrate that is potentially degradable, *U*_0_ (g) the amount that is undegradable, *T* (h) the lag before commencement of degradation, *c* (h^−1^) the fractional rate of fermentation, and *k* (h^−1^) is the fractional rate of passage out of the compartment [4].

If the profile is sigmoidal, first-order kinetics with a variable fractional degradation rate would account for substrate degradation and the France model (Equation (2)) can be fitted. Extent of degradation is then given by:

(6) $$E=S_{0}e^{-kT}\left(1-kI_{1}\right)/\left(S_{0}+U_{0}\right)$$

(7) $$=kS_{0}I_{2}/(S_{o}+U_{0})$$

where

$$I_{1}=\int_{T}^{\infty }\exp\left\{-\left[\left(c+k\right)\left(t-T\right)+d\left(\sqrt{t}-\sqrt{T}\right)\right]\right\}dt$$

$$I_{2}=\int_{T}^{\infty }e^{-kt}\left\{1-\exp\left[-c\left(t-T\right)-d\left(\sqrt{t}-\sqrt{T}\right)\right]\right\}dt$$

and *c* (h^−1^) is the constant portion of the fractional degradation rate and *d* (h^−0.5^) the coefficient of the variable portion. The integrals *I*_1_ and *I*_2_ are non-analytical and therefore have to be evaluated numerically [4].

If the profile is linear with an abrupt cut-off (i.e., a broken stick), zero-order kinetics with constant rate of degradation independent of substrate remaining can be assumed and a piecewise linear model fitted. The extent of degradation is then given by:

(8) $$E=\beta \left[\ln\left(k\beta^{-1}S_{0}+e^{kT}\right)-kT\right]/\left[k\left(S_{0}+U_{0}\right)\right]$$

where *β* (mL h^−1^) is the slope of the line fitting the ascending portion of the profile [22].

If the gas production profile is multiphasic, then the extent of degradation for each phase can be calculated by applying the appropriate equation, and the weighted extents summed to estimate overall extent of degradation. For example, if the profile resolves into two diminishing returns components (1 and 2) as in Equation (3), then Equation (5) can be independently applied to each of the two phases and the overall extent calculated as:

(9) $$E=\left(w_{1}E_{1}+w_{2}E_{2}\right)/\left(w_{1}+w_{2}\right)$$

where *w*_1_ and *w*_2_ are the relative weights assigned to the respective phases. If the profile resolves into a diminishing returns and a linear (with abrupt cut-off) component as in Equation (4), then Equations (5) and (8) respectively can be applied to the two phases and the overall extent calculated again using Equation (9). As an arbitrary rule of thumb, the asymptotic gas production values for the two phases (abrupt cut-off value if a phase is linear), viz. *A*_1_ and *A*_2_, can be adopted as the weights *w*_1_ and *w*_2_ respectively.

Thus, for the equine data considered herein, the extent of degradation of substrate in the hindgut can be calculated using Equations (5)–(9) if we assume faecal *inoculum* is representative of that region of the gastro-intestinal tract. Herein, when calculating extent of degradation, the amount of the incubated substrate that is potentially degradable *S*_0_ (g), the amount that is undegradable *U*_0_ (g), and the fractional rate of passage out of the compartment *k* (h^−1^), were assumed to be 0.538, 0.465 and 0.019, respectively, for all datasets [23].

### 2.4. Fitting and Evaluation of Models

Each of the four models (Equations (1)–(4)) was fitted by non-linear regression to the 25 gas production profiles using the NLIN procedure in the statistical software SAS [24]. Initial estimates of parameter values were obtained through visual inspection of the data.

Using various statistical tests, the models were evaluated for goodness-of-fit along with analysis of residuals. Mean square prediction error (MSPE) was calculated as the sum of the squared difference between predicted and observed values divided by the number of observations [25]. The accuracy factor (AF) index is a measure of the average deviation of a model’s predictions and is used as a simple index of the level of confidence in these predictions [26]. Agreement between model predictions and observations was further determined using the concordance correlation coefficient (CCC), a single statistic ranging between −1 (perfect disagreement) and +1 (perfect agreement) which contains both accuracy and precision indicators [27,28]. The Akaike information criterion (AIC) is a test for model selection which accounts for goodness-of-fit while penalizing for over-fitting, with the model resulting in the smallest AIC being the most appropriate [29].

The ability of each model to predict gas production without systematically over- or under-estimating was examined using the number of runs test and the Durbin–Watson (DW) test. The runs test examines a sequence of residuals for unusual groupings of positive or negative residuals and tests the null hypothesis that the arrangement of signs (+/−) is random, with too few runs indicating the presence of autocorrelation [30]. The DW test examines dependencies in the error terms by testing for correlations between a residual and the residuals immediately before and after it in the sequence. Compared to the runs test, the DW provides greater information regarding analysis of residuals by not only considering the sign of the residual but also its magnitude. The DW statistic (*D*), and upper (*D_u_*) and lower (*D_l_*) critical values, were calculated according to [30]. When *D* is less than the lower critical value *D_l_*, evidence of positive autocorrelation occurs, and when *D* is greater than the upper critical value *D_u_*, evidence of negative autocorrelation occurs.

## 3. Results

The ability of the Mitscherlich, and the other derived functions, to describe typical and atypical cumulative gas production profiles was assessed by fitting the four equations (Equations (1)–(4)) to 25 datasets. The profiles examined resulted from incubating forage using faecal *inoculum* from equines following the methodology of Theodorou et al. [2]. Using parameter estimates resulting from fitting these models to the gas production profiles, extents of substrate degradation were calculated and compared.

### 3.1. Fitting Behaviour

Of the 25 gas production profiles considered, 17 displayed atypical patterns, characterized by more than one phase, while the remaining 8 displayed typical monophasic patterns. No convergence issues were encountered when fitting the simple Mitscherlich (Equation (1)), double Mitscherlich (Equation (3)) and the Mitscherlich + linear (Equation (4)) to any of the datasets. The use of an “if than” statement concerning $t\;\geq T_{2}$ and its effect on *A*_2_ and *β* in SAS allowed both Equations (3) and (4) to revert to the simple Mitscherlich if that resulted in a better fit compared to the extended biphasic equations (i.e., when *A*_2_ = 0 in Equation (3) and *β* = 0 in Equation (4)). The France equation (Equation (2)) also encompasses the ability to revert to a simple Mitscherlich (Equation (1)) when *d* = 0. When fitted to the 25 gas production profiles, the France equation (Equation (2)) reverted to the simple Mitscherlich (Equation (1)) in four cases as the best fit for these gas production profiles was achieved when *d* = 0. Likewise, the double Mitscherlich (Equation (3)) reverted to the simple Mitscherlich (Equation (1)), i.e., *A*_2_ = 0, in five cases as a single Mitscherlich term described these profiles better than two Mitscherlich terms.

When fitting the France model (Equation (2)) to the atypical gas production curves, the convergence criteria had to be relaxed in order to reach successful convergence. When enforcing relaxed convergence criteria, Equation (2) was unable to converge for one of the 25 datasets. In order to achieve biologically meaningful parameters, lag time (*T*) and fractional rate constant (*c*) were constrained to be non-negative when fitting each model. Furthermore, in fitting Equation (2) a constraint was placed on parameter *d*, viz. d≥−2c√T to ensure the fractional rate of degradation remained non-negative [4].

### 3.2. Parameter Estimates and Fitted Gas Production Curves

**Figure 1:**
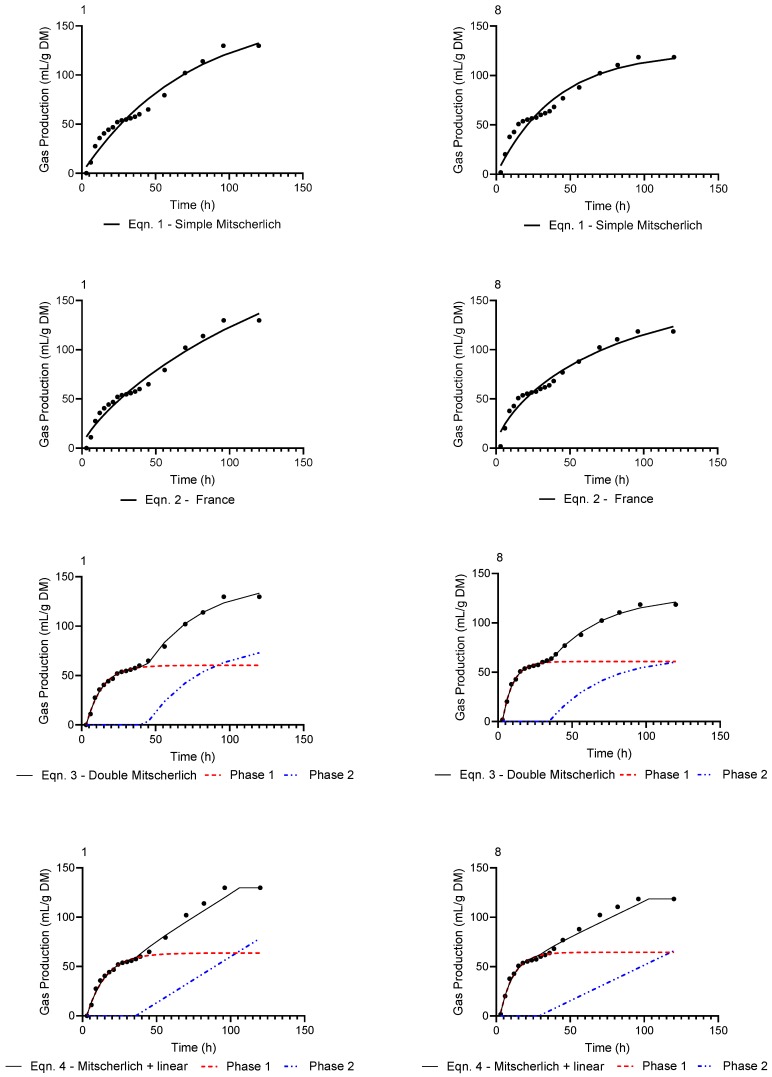
Observed (●) atypical gas production profiles and predicted curves resulting from fitting Equations (1)–(4) to Dataset 1 and 8 of Experiment 1.

Initial parameter estimates of lag time (*T*), asymptotic value (*A*) and slope (*β*) were determined by visual inspection of the gas production curves, while ranges for the fractional rate constants (*c* and *d*) were provided. The final parameter estimates resulting from fitting Equations (1)–(4) to Dataset 1 and 8 of Experiment 1 and Dataset 18 and 22 of Experiment 2 are presented in Table 2 and Table 3, respectively. The final estimates resulting from fitting Equations (1)–(4) to the remaining 21 datasets are given in the Supplementary Information section of this paper (Tables S2–S5). Using the parameter estimates in Table 2 and Table 3, the gas production profiles resulting from applying Equations (1)–(4) to Dataset 1 and 8 are shown in Figure 1. Both Dataset 1 and 8 show clear atypical biphasic gas production curves which are more faithfully represented by the double Mitscherlich (Equation (3)) and Mitscherlich + linear (Equation (4)) equations than the monophasic simple Mitscherlich (Equation (1)) or the France model (Equation (2)). Examining the four lower panels of Figure 1, the extent to which each phase contributes to the overall gas production curve of the double Mitscherlich (Equation (3)) and Mitscherlich + linear (Equation (4)) are clearly distinguishable.

**Figure 2:**
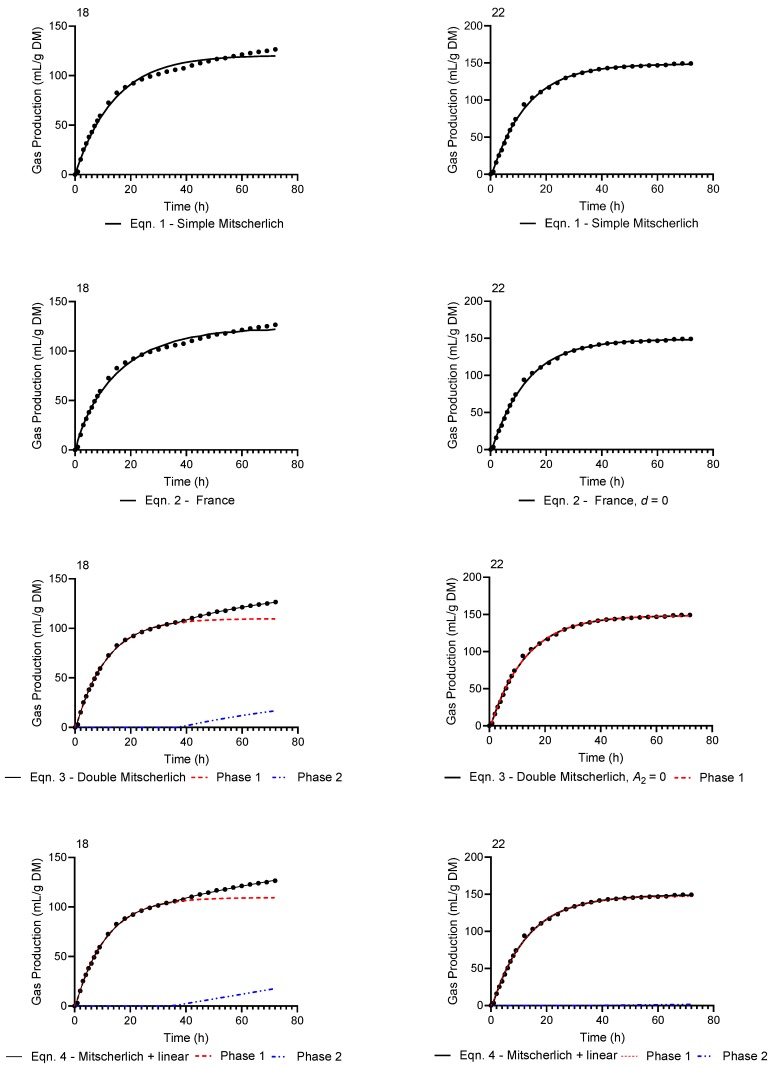
Observed (●) typical gas production profiles and predicted curves resulting from fitting Equations (1)–(4) to Dataset 18 and 22 of Experiment 2.

In contrast to Dataset 1 and 8, Dataset 18 and 22 of Experiment 2 display more typical monophasic gas production curves as shown in Figure 2. Again, examining the bottom four panels of Figure 2, the second phase of the biphasic models (viz. Equations (3) and (4)) is much less evident, with the second phase being entirely absent when fitting the double Mitscherlich (Equation (3)) to Dataset 22 as Equation (3) reverts to the simple Mitscherlich with *A*_2_ = 0. Additionally, when fitting the equation of France (Equation (2)) to Dataset 22, the best fit was achieved when *d* = 0, and thus Equation (2) reverted to a simple Mitscherlich (Equation (1)) when applied to this dataset. The gas production profiles resulting from fitting Equations (1)–(4) to the remaining datasets are shown in the Supplementary Information (Figures S1–S4).

### 3.3. Model Evaluation

Goodness-of-fit was assessed using four criteria, namely AIC, MSPE, CCC and AF. The goodness-of-fit values resulting from fitting each of the four models to the 25 datasets were averaged and the models ranked from 1 to 4 based upon their comparative performance with the other models for a given criterion. Individual models averaged goodness-of-fit values, along with their mean rank and the number of times the model ranked first or second under a given criterion, are presented in Table 4.

When fitted to the 25 datasets, the double Mitscherlich resulted in the smallest averaged AIC value (46.7 ± 2.7), followed by the Mitscherlich + linear (47.6 ± 5.2) and the France equation (66.6 ± 2.6), with the simple Mitscherlich (73.1 ± 2.5) resulting in the highest average AIC. Based upon AIC, the mean rank of the double Mitscherlich, Mitscherlich + linear, France and the simple Mitscherlich was 1.4, 1.7, 2.9 and 3.4, respectively, with the Mitscherlich + linear being ranked 1st or 2nd 25 times followed by the double Mitscherlich (23), France (5), and the simple Mitscherlich (2). The double Mitscherlich resulted in the lowest average MSPE (5.1 ± 0.5) followed by the Mitscherlich + linear (10.7 ± 1.7), France (20.9 ± 3.2), and the simple Mitscherlich (31.6 ± 5.4). In agreement with AIC, the double Mitscherlich resulted in the highest mean rank (1.3), followed by the Mitscherlich + linear (1.6), France (2.6) and simple Mitscherlich (3.4) with the Mitscherlich + linear ranking 1st or 2nd 25 times compared to the double Mitscherlich (22), France (5), and simple Mitscherlich (2).

Following the same trend as AIC and MSPE, the double Mitscherlich resulted in the highest CCC (0.996 ± 0.001), followed by the Mitscherlich + linear (0.994 ± 0.001), France (0.985 ± 0.003) and simple Mitscherlich (0.979 ± 0.004). Again, the double Mitscherlich yielded the best average rank of 1.4, followed by the Mitscherlich + linear (1.7), France (2.6) and simple Mitscherlich (3.5). The simple Mitscherlich was ranked 1st or 2nd in 5 of the 25 datasets and France in 9 of the 25, whilst the Mitscherlich + linear and double Mitscherlich were ranked 1st or 2nd in 25 and 22 datasets, respectively. On the basis of AF, the Mitscherlich + linear (1.17 ± 0.03) and double Mitscherlich (1.18 ± 0.03) outperformed both the France and simple Mitscherlich (with an averaged AF of 1.32 ± 0.04). The double Mitscherlich was ranked higher than the Mitscherlich + linear, 1.4 vs. 1.5, with both models being ranked 1st or 2nd 25 times. The simple Mitscherlich had a higher rank compared to the France, 2.8 vs. 3.2, with the simple Mitscherlich ranking 1st or 2nd 7 times compared to 3 times for the France equation.

In addition to goodness-of-fit, the runs test and DW test were used for the analysis of residuals. The results of these analyses are presented in Table 4. The runs test determined that too few runs occurred for all datasets when fitting the simple Mitscherlich and France equations. In comparison, runs of residuals were determined to be random in 18 and 6 of the 25 datasets when fitting the double Mitscherlich and Mitscherlich + linear, respectively. Using the DW test there was evidence of positive correlation of the residuals in all of the datasets that the simple Mitscherlich fitted successfully, and in all but one for the France model. In contrast, positive correlation was only found in 7 and 10 of the 25 datasets when fitting the double Mitscherlich and Mitscherlich + linear, with negative correlation being found in 16 and 11 of the 25 datasets, respectively.

### 3.4. Extent of Degradation

Extent of substrate degradation, calculated from the parameter estimates resulting from fitting the four models to the 25 gas production profiles, is presented in Table 5. When calculating extent of degradation using the biphasic equations, viz. Equations (3) and (4), the relative weights in which each phase contributed to the overall extent of substrate degradation need to be incorporated. Using the double Mitscherlich (Equation (3)) the weights of each phase were simply assumed to be their respective asymptotic gas production values for each phase, *A*_1_ and *A*_2_. For the Mitscherlich + linear, the weight of the first phase was its respective asymptotic value (*A*_1_), while the weight of the second (linear) phase was calculated as the amount of gas produced over the course of this linear segment (i.e., multiplying its slope by the duration of the linear segment). The duration of the linear segment was the difference between time at which the abrupt cut-off value occurred and time at which the linear portion commenced (i.e., *T*_2_, the lag time). The abrupt cut-off was determined in two ways. In Experiment 1, Dataset 1–14, abrupt cut-off values were assumed to occur at the intersection between the linear segment of the equation and the apparent plateau in gas production, which visually occurred between the last two data points. In Experiment 2, Dataset 15–17, a plateau in the observed data was not evident, therefore the abrupt cut-off value was set to the end of incubation. Finally, in Experiment 2, Dataset 18–25, a plateau was eventually reached with the linear segments being horizontal or near horizontal, and abrupt cut-off values were again set to the end of incubation.

Although substrate was primarily grass hay, or a grass hay mix, the range of calculated extent of degradation varied widely, from a minimum of 12.7% to a maximum of 44.2%. The wide range in extent of degradation values can be attributed to the use of parameter estimates from a model that fits a given gas production profile poorly. Therefore, Table 5 includes an indicator of which model fitted the particular dataset values best, based upon AIC. Fitting these four models to 25 datasets that encompass both typical and atypical gas production curves resulted in the double Mitscherlich being the best fitting in 15 of these datasets, the Mitscherlich + linear 8, simple Mitscherlich 2 and France 0. Given that extent of degradation is determined using parameter estimates obtained by fitting these models to a dataset, the importance of model fit in calculating extent of degradation is apparent.

## 4. Discussion

Gas production profiles generated from incubating a substrate with either ruminal or faecal *inocula* have been widely used to provide information regarding the degradability of forages and supplementary feeds in both ruminants and non-ruminants [3,5,6,10,11,12,13,14]. Typical shapes of these profiles range from diminishing returns to strongly sigmoidal [3]. Various models, e.g., Mitscherlich, Michaelis-Menten, Gompertz and logistic, have been proposed to describe these curves, including generalised models such as Richards and that of France which are able to accommodate both diminishing returns and sigmoidal behaviour [4,31,32,33]. Deriving these models on the basis of substrate degradation rather than amount of gas produced permits the generation of fermentation kinetic parameters [3]. By fitting these models to gas production profiles, such parameters (e.g., fractional rate of degradation and lag time) can be estimated. These model-derived parameters have been used in conjunction with information regarding substrate degradability and digesta passage rate to calculate extent of substrate degradation in the rumen [3,4,5,6,34]. This method has been successfully applied to typical monophasic sigmoidal and diminishing returns gas profiles to evaluate substrates based upon the extent of their degradability [3,5,6,8,9,10,11,12,13,14,34].

In addition to the typical sigmoidal and diminishing return patterns displayed by gas production profiles, atypical multiphasic curves have been reported [15,16,19,20,35]. Although multiphasic gas production curves have been described by both Groot et al. [15] and Wang et al. [36], proposed models are based upon the amount of gas produced, rather than the amount of substrate degraded, resulting in the model being unable to link the gas production technique to animal performance [3].

### 4.1. Profile Shapes and Associated Parameters

The diminishing returns behaviour described by the simple Mitscherlich is a result of the interaction between the constant fractional degradation rate (*c*) and the amount of degradable substrate (*S*) available for fermentation. The amount of degradable substrate available is time dependent with the maximal value occurring at time zero (*S*_0_). The instantaneous rate of degradation is calculated by multiplying the constant fractional degradation rate (*c*) by the amount of degradable substrate (*S*) at time *t* [3]. As the fractional degradation rate is constant, and *S* is maximal at the commencement of the incubation, instantaneous rate of degradation is maximal at the start of incubation, following a lag period if present. As fermentation progresses, the amount of degradable substrate decreases while the fractional rate of degradation remains constant. Therefore, the instantaneous rate of degradation declines continuously, from its maximum at the start of the incubation until it finally reaches zero due to available fermentable material being exhausted. When fermentation ceases, due to a lack of degradable substrate, the instantaneous rate of degradation becomes zero, with no additional gas production occurring, having reached an upper asymptote. Therefore, the characteristic diminishing returns pattern of the simple Mitscherlich describes a scenario whereby rate of fermentation, and thus gas production, is initially at a maximum and continuously decreases, as a function of time, until an asymptote is reached.

Unlike the simple Mitscherlich, the France model is capable of describing both diminishing returns and sigmoidal behaviour. This is achieved by assuming that fractional rate of degradation can vary with time. Depending on the values of the fractional rate constants, viz. *c* and *d* in this manuscript, the fractional rate of degradation can remain constant, decrease or increase with time [4]. In the France model, when the fractional rate of degradation is constant diminishing return type behaviour is described (as in the simple Mitscherlich). None of the gas production profiles examined in this study showed clear sigmodal behaviour and therefore the flexibility of the France model in its ability to describe both diminishing returns and sigmoidal shapes was not demonstrated. When describing sigmoidal behaviour, initially the rate of degradation increases resulting in exponential-type behaviour. As it continues to increase, a point of inflexion occurs whereby the rate reaches its maximal value. Following inflexion, the rate of degradation decreases resulting in diminishing returns behaviour with an asymptote being approached.

Of the 25 datasets examined in this study, in all but three the substrate was ground and passed through a 1 mm screen prior to incubation, with the remaining three being chopped (length not reported). A clear increase in gas production, and associated increase in extent of substrate degradation, is observed when comparing the ground (Dataset 18–25) with the chopped substrates (Dataset 15–17) of Experiment 2 (see Supplementary Information Figure S3 vs. Figure S4 and Table 5 for associated gas production profiles and extent of degradation values, respectively). However, 17 of these datasets, including both ground and chopped substrates, are atypical in nature and exhibit a second phase of gas production. In these 17 datasets the first phase is well described by the simple Mitscherlich whereby following a lag, rate of degradation and thus gas production are initially at their maximum and continuously decrease until an asymptote is approached. Following this first phase, a second phase occurs. This second phase can show either diminishing returns or a linear pattern. These phases might be attributed to differences in chemical or nutritional fractions of the feedstuffs [16]. Phase 1 may represent the gas produced from the fermentation of sugars or a soluble readily fermentable fraction, while the second phase consists of gas produced from the fermentation of structural carbohydrates or an insoluble potentially fermentable fraction [35,37]. Alternatively, the occurrence of the second gas production phase can potentially be attributed to chemical or structural barriers implicit in the substrate that must be overcome in order to continue degradation [15]. Furthermore, the possibility of microbial turnover in batch cultures, and the small amount of gas produced from ‘self-fermentation’ may add to the second phase of gas production [35,38]. Many other factors may influence the profile shape including: inter-animal variability, ration of the donor of the microbial *inoculum*, length of time the donor animal was adapted to the ration, time of day the *inoculum* was collected, ruminal vs. faecal sources of *inoculum* and frozen vs. fresh *inoculum* [5,19,35]. This leads to the conclusion that these factors may have the potential to influence microbial diversity and abundance in the *inoculum*, which in turn influences fermentative ability and by extension influences gas production.

### 4.2. Extent of Degradation

The ability to describe typical diminishing returns and sigmoidal gas production profiles using a variety of models (e.g., Mitscherlich, Michaelis-Menten, logistic, Gompertz and France) is well established [3,34,36]. However, the description of atypical multiphasic gas production curves is much less established, particularly how to link the in vitro gas production technique to the extent of degradation in the animal. Groot et al. [15] proposed a model that fitted multiphasic profiles using two or more generalised rectangular hyperbolae. Applying this model, some authors have estimated the amount of gas produced and rate of gas production of various feedstuffs [35,39]. Likewise, Wang et al. [36] described single- and multi-phase gas production curves using logistic-exponential equations. However, in these studies differences in feedstuffs were identified on the basis of the amount of gas produced rather than on criteria linked to animal performance.

Two biphasic models are presented in this paper that make use of a simple Mitscherlich term when describing the first phase of gas production and a second phase comprising either an additional Mitscherlich or a simple linear term with abrupt cut-off. Depending on the nature of the profile, both the double Mitscherlich (Equation (3)) and Mitscherlich + linear (Equation (4)) fitted the atypical datasets well, resulting in parameter values that can be used to calculate and compare the extent of degradation of respective substrates and substrate treatments. For example, three of the datasets examined in this study (Dataset 15–17) encompass chopped hay treated with increasing levels of an enzyme (0, 0.75 or 3.7 mL enzyme per kg DM hay, respectively). The Mitscherlich + linear fitted these datasets the best and using the associated parameter values, extent of substrate degradation was demonstrated to increase with increasing levels of enzymatic treatment, viz. 38.4%, 38.9% and 42.0% for Dataset 15–17, respectively. When fitting the Mitscherlich + linear to these datasets, it was assumed that following the linear trend an abrupt cut-off is reached (i.e., an asymptote is reached) and gas production ceases. This can be observed by inspecting Dataset 1 and 8 in Figure 1 whereby gas production ceases to increase between the last two data points. In comparison, examining Dataset 15–17 (see Supplementary Information, Figure S3), visually there appears to be potential for further gas production as an asymptote, in the form of an abrupt cut-off following the linear segment, has yet to be reached. If the linear trend continues after the 76 h incubation period, there is potential for continued substrate degradation and associated gas production. Therefore, extent of substrate degradation calculated using the Mitscherlich + linear would be an underestimate if fermentation continued beyond the 76 h incubation period used to generate these gas production profiles.

As previously mentioned, the extent of degradation calculated using the fermentation kinetic parameters generated from applying the four proposed models to 25 datasets ranged widely from a minimum of 12.7% to a maximum of 44.2%. This wide range in values can partially be attributed to the use of parameter values from a model that fits a particular gas production profile poorly. Even in a given dataset, large variation in calculated extent of degradation values existed. For example, in Dataset 3, extent of degradation using the France model was 12.7% compared to 37.5% with the Mitscherlich + linear. These findings are in contrast with those of Dhanoa et al. [34] whereby the model being applied (generalised Mitscherlich, simple Mitscherlich, generalised Michaelis-Menten, simple Michaelis-Menten, Gompertz and logistic) had very little effect on extent of degradation values. However, the gas production profiles of Dhanoa et al. [34] using mixed rumen microorganisms as the *inoculum* were all monophasic in nature and therefore reasonably well described by the aforementioned models. Indeed, when examining the eight typical gas production profiles of this manuscript, Dataset 18–25, there was very little difference in extent of degradation in a given dataset regardless of model applied. When comparing the standard deviation of extent of degradation determined by the four models when applied to the same typical gas production dataset, Dataset 22 had the lowest value of 0.1% while Dataset 18 had the highest deviation at 3.1%. Examining Dataset 22, the simple Mitscherlich fitted the dataset best with an associated extent of degradation of 42.9%. Both the double Mitscherlich and France models reverted to the simple Mitscherlich, as the simple Mitscherlich fitted this dataset better than their generalized forms, and therefore were in agreement with an extent of degradation of 42.9%. The Mitscherlich + linear was also in agreement with this value, 42.8%. In contrast, examining the 17 atypical biphasic gas production profiles, the model applied had considerable ramifications on calculated extent of degradation. In these datasets, the lowest standard deviation of extent of degradation between the four models when applied to a single dataset occurred in Dataset 12 at 3.4% while the largest deviation occurred in Dataset 3 at 10.1%. In the atypical profile of Dataset 12, the double Mitscherlich fitted the gas production profile the best with a calculated extent of degradation of 30.2%, the simple Mitscherlich, France and Mitscherlich + linear overestimated the extent of degradation, viz. 33.4%, 32.6% and 38.2%, respectively. Overall, this discrepancy in extent of degradation values for a given dataset can be attributed to fitting a monophasic equation (viz. Equations (1) and (2)) to a distinctly biphasic profile, or fitting a linear term to a non-linear segment, resulting in poor kinetic parameter estimates and by extension extent of degradation values.

It is important to note that when calculating extent of degradation, the value of *S*_0_, the amount of incubated substrate that is potentially degradable, was taken from the literature. This value was set to 0.538 regardless of dataset and the associated substrate represented by that dataset. The value of 0.538 is the apparent in vivo dry matter digestibility, using ponies, of ground and pelleted hay consisting of a 50:50 mix of Lucerne hay and Cocksfoot hay [23]. When performing the gas production technique of Theodorou et al. [2], the potentially undegradable fraction of the substrate (*U*_0_) can be obtained by weighing the residual matter after gas production has ceased. Likewise, the potentially degradable value (*S*_0_) can be calculated by subtracting *U*_0_ from the quantity of substrate initially incubated. However, these values were not available at the time of this current study and a constant value was assumed. Therefore, greater differences in calculated values of extent of degradation should be expected as *S*_0_ and *U*_0_ will vary between substrates, substrate composition and the treatment received.

## 5. Conclusions

Two models, a double Mitscherlich and Mitscherlich + linear with abrupt cut-off, were proposed and derived to describe atypical gas production patterns characterized by two distinct phases of gas production. The models fitted these atypical curves well and due to their hybrid nature are also able to describe typical monophasic gas production profiles through their ability to revert to a simple Mitscherlich. These models contain kinetic parameters that can be used to calculate extent of substrate degradation using relatively simple equations. Given that extent of degradation is linked to nutrient supply, these models provide useful information regarding the evaluation of feedstuffs using in vitro methods [34,40].

## Figures and Tables

**Table 1:** Details of the eleven treatments used in Experiment 2.

**Control and Enzyme Treatments**		
Dataset-Substrate-*Inoculum*	Substrate (Chopped)	*Inoculum* from
**15-C-I1**	**C** Grass hay untreated (same as ponies were fed).	**I1** Ponies fed control diet consisting of grass hay fed at 100%
**16-E1-I2**	**E1** Grass hay treated with enzyme 0.75 mL/kg DM hay	**I2** Ponies fed grass hay treated with enzyme 0.75 mL/kg DM hay
**17-E2-I3**	**E2** Grass hay treated with enzyme 3.75 mL/kg DM hay	**I3** Ponies fed grass hay treated with enzyme 3.75 mL/kg DM hay
**Other treatments**		
Dataset-Substrate-*Inoculum*	Substrate (Ground to pass 1 mm screen)	*Inoculum* from
**18-A-I4**	**A** Grass hay (50%) + alfalfa (50%)	**I4** Ponies fed 25% alfalfa and 75% grass hay (dry matter (DM) basis). Not supplemented with yeast.
**19-A1-I4**	**A1** Grass hay (75%) + alfalfa (25%)	
**20-B-I5**	**B** Grass hay (50%) + alfalfa (50%) + Yeast (0.011 g)	**I5** Ponies fed 25% alfalfa and 75% grass hay (DM basis). Supplemented daily with 30 g yeast per pony was mixed with alfalfa.
**21-B1-I5**	**B1** Grass hay (75%) + alfalfa (25%) + Yeast (0.011 g)	
**22-C-I6**	**C** Grass hay (50%) + concentrate (50%)	**I6** Ponies fed 25% concentrate and 75% grass hay (DM basis). Not supplemented with yeast.
**23-C1-I6**	**C1** Grass hay (75%) + concentrate (25%)	
**24-D-I7**	**D** Grass hay (50%) + concentrate (50%) + Yeast (0.011 g)	**I7** Ponies fed 25% concentrate and 75% grass hay (DM basis). Supplemented daily with 30 g yeast per pony mixed with concentrate.
**25-D1-I7**	**D1** Grass hay (75%) + concentrate (25%) + Yeast (0.011 g)	

**Table 2:** Parameter estimates obtained by fitting Equations (1)–(4) to Dataset 1 (laminitis) and 8 (clinically normal) from Experiment 1. An asterisk (*) denotes the equation that resulted in the best fit, based on AIC, to a particular dataset.

	Simple Mitscherlich (Equation (1))		France (Equation (2))		Double Mitscherlich (Equation (3)) ^‡^		Mitscherlich + linear (Equation (4)) ^§^	
	Dataset 1	Dataset 8	Dataset 1	Dataset 8	Dataset 1	Dataset 8	Dataset 1	Dataset 8
*A*	163.5	123.7	246.8	159.5	60.3, 84.3 *	60.8, 66.8 *	63.7	64.6
*c*	0.014	0.025	0.005	0.008	0.093, 0.026 *	0.140, 0.027 *	0.078	0.111
*T*	0	0	0	0	3.1, 43.4 *	2.8, 34.6 *	2.8, 35.9	2.5, 28.7
*d*			0.019	0.049				
*β*							0.936	0.723

^‡^ The two scale parameters of this equation are entered under *A* in the order *A*_1_, *A*_2_ in this table. Likewise, the two rate parameters under *c* in the order *c*_1_, *c*_2_, and the two lags under *T* in the order *T*_1_, *T*_2_. ^§^ The two lag parameters of this equation are entered under *T* in the order *T*_1_, *T*_2_.

**Table 3:** Parameter estimates obtained by fitting Equations (1)–(4) to Dataset 18 (50% grass hay + 50% alfalfa) and 22 (50% grass hay + 50% concentrate), from Experiment 2. An asterisk (*) denotes the equation that resulted in the best fit, based on AIC, to a particular dataset.

	Simple Mitscherlich (Equation (1))		France (Equation (2))		Double Mitscherlich (Equation (3)) ^‡^		Mitscherlich + linear (Equation (4)) ^§^	
	Dataset 18	Dataset 22	Dataset 18	Dataset 22	Dataset 18	Dataset 22	Dataset 18	Dataset 22
*A*	120.6	148.7 *	122.9	148.7 ^†^	109.7, 38.7	148.7 ^Ψ^	109.6 *	147.7
*c*	0.071	0.078 *	0.057	0.078 ^†^	0.090, 0.016	0.078 ^Ψ^	0.091 *	0.079
*T*	0	0.5 *	0	0.5 ^†^	0.3, 37.2	0.5 ^Ψ^	0.3, 34.8 *	0.5, 33.1
*d*			0.037					
*β*							0.473 *	0.040

^‡^ The two scale parameters of this equation are entered under *A* in the order *A*_1_, *A*_2_ in this table. Likewise, the two rate parameters under *c* in the order *c*_1_, *c*_2_, and the two lags under *T* in the order *T*_1_, *T*_2_. ^§^ The two lag parameters of this equation are entered under *T* in the order *T*_1_, *T*_2_. ^†^ Best fit by France, Equation (2), achieved with *d* = 0, therefore reverting to a simple Mitscherlich, viz. Equation (1). ^Ψ^ Best fit by double Mitscherlich, Equation (3), achieved with *A*_2_ = 0, therefore reverting to a simple Mitscherlich, viz. Equation (1).

**Table 4:** Goodness-of-fit and analysis of residuals from fitting the four equations to the 25 datasets of Experiments 1 and 2.

Criteria	Model			
	Simple Mitscherlich (Equation (1))	France (Equation (2))	Double Mitscherlich (Equation (3))	Mitscherlich + Linear (Equation (4))
Akaike information criterion (AIC)				
Average (±SE)	73.1 (2.5)	66.6 (2.6)	46.7 (2.7)	47.6 (5.2)
Mean rank	3.4	2.9	1.4	1.7
Number of times a model ranked 1st or 2nd	2	5	22	25
Mean square prediction error (MSPE)				
Average (±SE)	31.6 (5.4)	20.9 (3.2)	5.1 (0.5)	10.7 (1.7)
Mean rank	3.4	2.6	1.3	1.6
Number of times a model ranked 1st or 2nd	2	5	22	25
Concordance correlation coefficient (CCC)				
Average (±SE)	0.979 (0.004)	0.985 (0.003)	0.996 (0.001)	0.994 (0.001)
Mean rank	3.5	2.6	1.4	1.7
Number of times a model ranked 1st or 2nd	5	9	22	25
Accuracy factor (AF)				
Average	1.32 (0.04)	1.32 (0.04)	1.18 (0.03)	1.17 (0.03)
Mean rank	2.8	3.2	1.4	1.5
Number of times a model ranked 1st or 2nd	7	3	25	25
Number of runs test				
Too few runs	25	24	7	19
Runs are random	0	0	18	6
Durbin–Watson (DW) test				
Positive correlation	25	23	7	10
No evidence	0	1	2	4
Negative correlation	0	0	16	11

**Table 5:** Calculated extent of degradation (%) from the 14 datasets of Experiment 1 displaying atypical gas production profiles and the 11 datasets of Experiment 2 displaying both typical and atypical profiles. An asterisk (*) denotes the equation that resulted in the best fit, based on AIC, to a particular dataset.

Dataset	Visual Curve Pattern	Simple Mitscherlich (Equation (1))	France (Equation (2))	Double Mitscherlich (Equation (3))	Mitscherlich + Linear (Equation (4))
1	Atypical	22.7	15.5	25.5 *	33.9
2	Atypical	34.3	28.5	35.1 *	40.0
3	Atypical	25.4	12.7	25.1 *	37.5
4	Atypical	26.7	-	25.0 *	35.6
5	Atypical	31.1	21.8	29.1 *	42.0
6	Atypical	30.3	21.7	30.0 *	38.0
7	Atypical	34.9	28.6	32.7 *	39.7
8	Atypical	30.4	24.4	30.0 *	38.0
9	Atypical	23.4	16.0	33.2 *	36.8
10	Atypical	22.4	19.0	24.2 *	34.9
11	Atypical	36.3	32.9	29.1 *	39.6
12	Atypical	33.4	32.6	30.2 *	38.2
13	Atypical	25.9	22.0	28.1 *	34.5
14	Atypical	33.3	31.3	25.1 *	37.3
15	Atypical	31.6	31.6	31.6 ^Ψ^	38.4 *
16	Atypical	34.2	23.5	34.2 ^Ψ^	38.9 *
17	Atypical	35.5	19.5	35.5 ^Ψ^	42.0 *
18	Typical	42.4	41.6	35.9	41.9 *
19	Typical	42.1	40.5	36.8 *	42.8
20	Typical	43.3 *	43.3 ^†^	43.3 ^Ψ^	43.0
21	Typical	43.2	43.2 ^†^	41.2	42.9 *
22	Typical	42.9 *	42.9 ^†^	42.9 ^Ψ^	42.8
23	Typical	42.5	42.3	41.9	43.0 *
24	Typical	44.2	44.2 ^†^	43.1	43.8 *
25	Typical	43.0	43.1	41.2	42.8 *

^†^ Best fit by France, Equation (2), achieved when *d* = 0, therefore reverting to a simple Mitscherlich, viz. Equation (1). ^Ψ^ Best fit by double Mitscherlich, Equation (3), achieved with *A*_2_ = 0, therefore reverting to a simple Mitscherlich, viz. Equation (1).

## Supplementary Materials

The following are available online at https://www.mdpi.com/2076-2615/10/2/308/s1, **Table S1**: Observed Gas Production Values of Datasets 1–25 from Experiment 1 and 2; **Table S2**: Final parameter estimates from fitting the simple Mitscherlich (Eqn. 1) to Dataset 1–25; **Table S3**: Final parameter estimates from fitting the France model (Eqn. 2) to Dataset 1–25; **Table S4**: Final parameter estimates from fitting the double Mitscherlich model (Eqn. 3) to Dataset 1–25; **Table S5**: Final parameter estimates from fitting the Mitscherlich + linear (Eqn. 4) to Dataset 1–25. **Figure S1**: Observed (●) and predicted gas production profiles resulting from fitting Equations (1)–(4) to Dataset 1–7, horses displaying clinical signs of laminitis, from Experiment 1; **Figure S2**: Observed (●) and predicted gas production profiles resulting from fitting Equations (1)–(4) to Datasets 8–14, clinically normal horses, from Experiment 1; **Figure S3**: Observed (●) and predicted gas production profiles resulting from fitting Equations (1)–(4) to Dataset 15–17, datasets exhibiting atypical dual-phase gas production curves, from Experiment 2. **Figure S4:** Observed (●) and predicted gas production profiles resulting from fitting Equations (1)–(4) to Dataset 18–25, datasets exhibiting typical single-phase gas production curves, from Experiment 2.
