# Supplementary material for: Models Based on the Mitscherlich Equation for Describing Typical and Atypical Gas Production Profiles Obtained from In Vitro Digestibility Studies Using Equine Faecal Inoculum
Source: Animals (Basel). 2020 Feb 17;10(2):308. doi: 10.3390/ani10020308 (PMC7070440; doi:10.3390/ani10020308)
Supplement: Supplementary file 1 [file animals-10-00308-s001.pdf]

Table S1. Observed Gas Production Values of Datasets 1– 25 from Experiment 1 and 2.

Datasets 1 – 7

| | Observed Gas Production (mL/g DM) | | | | | | |
|---|---|---|---|---|---|---|---|
| Time (h) | 1 | 2 | 3 | 4 | 5 | 6 | 7 |
| 3 | 0 | 1.738 | 0 | 0.522 | 0.029 | 0.956 | 3.091 |
| 6 | 11.02 | 18.54 | 15.03 | 18.69 | 23.88 | 22.94 | 26.25 |
| 9 | 27.5 | 38.6 | 28.93 | 33.47 | 37.5 | 39.54 | 41.95 |
| 12 | 35.75 | 47.69 | 35.44 | 39.64 | 43.23 | 48.58 | 49.75 |
| 15 | 40.61 | 51.63 | 39.26 | 43.2 | 46.44 | 55.88 | 56.34 |
| 18 | 44.25 | 53.72 | 40.65 | 45.89 | 47.83 | 60.14 | 61.98 |
| 21 | 46.77 | 55.26 | 42.3 | 45.89 | 50.78 | 61.01 | 62.79 |
| 24 | 52.06 | 57.75 | 44.13 | 45.98 | 53.29 | 61.53 | 63.95 |
| 27 | 53.79 | 59.66 | 45.78 | 46.16 | 55.03 | 62.48 | 63.95 |
| 30 | 54.66 | 60.85 | 47.52 | 46.3 | 57.19 | 62.86 | 65.74 |
| 33 | 55.88 | 63.46 | 48.82 | 47.08 | 59.36 | 64.25 | 67.64 |
| 36 | 57.52 | 65.48 | 50.73 | 47.69 | 61.01 | 66.86 | 68.71 |
| 39 | 60.04 | 67.45 | 52.73 | 48.65 | 62.57 | 69.46 | 70.88 |
| 45 | 64.98 | 72.53 | 59.07 | 51.6 | 67.6 | 78.76 | 77.65 |
| 56 | 79.47 | 80.12 | 68.11 | 58.04 | 80.18 | 94.92 | 88.4 |
| 70 | 102.1 | 89.21 | 85.83 | 78.72 | 91.03 | 109.5 | 100.1 |
| 82 | 113.9 | 98.14 | 96.95 | 92.37 | 103.1 | 119.1 | 109.1 |
| 96 | 129.8 | 107.6 | 107.8 | 106.4 | 109.6 | 127.5 | 113.6 |
| 120 | 129.8 | 107.6 | 107.8 | 106.4 | 109.6 | 127.5 | 113.6 |

Datasets 8 – 14

| | Observed Gas Production (mL/g DM) | | | | | | |
|---|---|---|---|---|---|---|---|
| Time (h) | 8 | 9 | 10 | 11 | 12 | 13 | 14 |
| 3 | 1.819 | 0 | 0 | 0 | 0 | 0.348 | 0 |
| 6 | 20.19 | 7.118 | 4.773 | 6.081 | 3.82 | 14.33 | 8.069 |
| 9 | 37.87 | 27.86 | 20.05 | 26.41 | 23.61 | 32.12 | 27.42 |
| 12 | 42.81 | 35.16 | 28.46 | 35.01 | 32.64 | 34.9 | 39.22 |
| 15 | 50.86 | 38.45 | 34.97 | 38.57 | 39.32 | 40.8 | 50.41 |
| 18 | 53.64 | 38.89 | 36.62 | 38.57 | 40.1 | 43.84 | 55.01 |
| 21 | 55.25 | 38.89 | 37.05 | 38.57 | 40.1 | 43.84 | 55.99 |
| 24 | 56.52 | 38.89 | 38.79 | 38.57 | 41.75 | 44.88 | 57.37 |
| 27 | 57.48 | 40.97 | 38.79 | 39.03 | 43.14 | 46.87 | 58.42 |
| 30 | 60.16 | 43.4 | 41.39 | 40.25 | 45.57 | 48.78 | 59.28 |
| 33 | 61.81 | 44.44 | 42.95 | 40.65 | 46.53 | 50.52 | 59.63 |
| 36 | 63.8 | 48.61 | 47.38 | 43.78 | 51.04 | 57.29 | 63.97 |
| 39 | 68.14 | 53.65 | 51.98 | 47.25 | 55.9 | 64.32 | 68.83 |
| 45 | 76.97 | 58.85 | 56.57 | 48.99 | 58.77 | 71.18 | 70.13 |
| 56 | 87.89 | 70.49 | 67.25 | 52.38 | 62.93 | 83.25 | 75.34 |
| 70 | 102.3 | 85.59 | 81.65 | 56.81 | 67.79 | 95.4 | 85.75 |
| 82 | 110.6 | 96.18 | 92.5 | 61.24 | 73.17 | 105.2 | 95.29 |

| | | | | | | | |
|---|---|---|---|---|---|---|---|
| 96 | 118.6 | 107.6 | 104.0 | 69.06 | 80.81 | 114.8 | 106 |
| 120 | 118.6 | 107.6 | 104.0 | 69.06 | 80.81 | 114.8 | 106 |

Datasets 15 – 20

| | Observed Gas Production (mL/g DM) | | | | | |
|---|---|---|---|---|---|---|
| Time (h) | 15 | 16 | 17 | 18 | 19 | 20 |
| 0 | 0 | 0 | 0 | 0 | 0 | 0 |
| 1 | 0.91 | 1.213 | 1.972 | 2.882 | 2.882 | 0.758 |
| 2 | 1.972 | 2.578 | 4.246 | 15.32 | 17.14 | 14.86 |
| 3 | 5.156 | 5.915 | 7.431 | 25.18 | 31.09 | 26.09 |
| 4 | 7.583 | 9.099 | 10.16 | 31.39 | 38.82 | 32 |
| 5 | 9.554 | 11.53 | 12.44 | 38.07 | 44.44 | 38.82 |
| 6 | 11.07 | 13.5 | 14.26 | 42.92 | 49.14 | 45.35 |
| 7 | 13.04 | 15.77 | 16.23 | 49.14 | 54.6 | 51.26 |
| 8 | 14.41 | 17.14 | 17.59 | 54.45 | 59.6 | 58.54 |
| 9 | 15.77 | 18.81 | 19.41 | 59.45 | 63.54 | 64.76 |
| 12 | 17.9 | 22.14 | 23.96 | 72.64 | 74.92 | 82.96 |
| 15 | 19.41 | 24.27 | 27.45 | 82.65 | 87.36 | 97.52 |
| 18 | 21.08 | 26.84 | 30.63 | 88.27 | 93.72 | 104.6 |
| 21 | 23.05 | 28.97 | 33.52 | 92.36 | 98.43 | 110 |
| 24 | 24.87 | 30.79 | 35.94 | 96.3 | 102.4 | 113.7 |
| 27 | 26.24 | 32.76 | 37.61 | 99.18 | 106.3 | 117.5 |
| 30 | 27.3 | 34.12 | 38.22 | 101.6 | 109.8 | 120.3 |
| 33 | 28.82 | 36.09 | 38.22 | 104 | 114 | 122.5 |
| 36 | 29.72 | 37.31 | 38.22 | 105.9 | 117.2 | 124.4 |
| 39 | 31.85 | 38.52 | 39.43 | 107.4 | 119.5 | 125.9 |
| 42 | 32.91 | 40.34 | 40.8 | 110.3 | 122.2 | 126.8 |
| 45 | 34.88 | 41.71 | 42.62 | 112.7 | 125 | 127.8 |
| 48 | 35.49 | 43.53 | 44.44 | 114.5 | 127.5 | 128.5 |
| 51 | 37 | 45.35 | 46.1 | 116.8 | 129.7 | 129.1 |
| 54 | 38.52 | 47.01 | 47.92 | 117.8 | 130.6 | 129.5 |
| 57 | 40.8 | 47.62 | 50.5 | 119.7 | 132.2 | 130 |
| 60 | 42.31 | 50.05 | 52.63 | 121.3 | 133.6 | 130.7 |
| 63 | 44.28 | 50.5 | 54.75 | 122.8 | 135.1 | 131.6 |
| 66 | 46.1 | 53.99 | 57.18 | 124.1 | 135.9 | 132.7 |
| 69 | 47.92 | 55.36 | 59.45 | 125.1 | 137.4 | 132.5 |
| 72 | 49.14 | 56.72 | 61.42 | 126.5 | 138.8 | 133 |

Datasets 21 – 25

| | Observed Gas Production (mL/g DM) | | | | |
|---|---|---|---|---|---|
| Time (h) | 21 | 22 | 23 | 24 | 25 |
| 0 | 0 | 0 | 0 | 0 | 0 |
| 1 | 0.455 | 3.336 | 3.64 | 1.82 | 1.972 |
| 2 | 15.01 | 15.92 | 17.59 | 12.74 | 13.35 |
| 3 | 32 | 25.33 | 29.72 | 26.39 | 29.72 |
| 4 | 40.19 | 32.61 | 37.31 | 37.91 | 40.19 |
| 5 | 46.56 | 41.86 | 44.44 | 48.99 | 48.23 |
| 6 | 51.87 | 50.5 | 50.5 | 58.09 | 54.9 |
| 7 | 58.09 | 59.45 | 56.57 | 65.67 | 60.66 |
| 8 | 63.7 | 67.03 | 61.72 | 72.64 | 66.58 |
| 9 | 70.37 | 74.31 | 67.49 | 77.95 | 72.04 |
| 12 | 89.02 | 94.03 | 83.41 | 96.61 | 90.99 |
| 15 | 105.4 | 103.1 | 94.63 | 108.9 | 103.1 |
| 18 | 111.6 | 110.7 | 101.2 | 116.5 | 110.9 |
| 21 | 117.7 | 116.9 | 107.1 | 122.5 | 117.7 |
| 24 | 121.8 | 123 | 112.4 | 128.2 | 123.1 |
| 27 | 125.7 | 129.7 | 118.9 | 131.3 | 127.7 |
| 30 | 127.8 | 133.6 | 122.2 | 132.7 | 129.4 |
| 33 | 130.7 | 136.8 | 125 | 134.2 | 131.6 |
| 36 | 133.9 | 139.2 | 128.2 | 135.4 | 133.5 |
| 39 | 136.5 | 141.6 | 130.9 | 136.8 | 135.3 |
| 42 | 138.5 | 143 | 132.7 | 139.1 | 138.5 |
| 45 | 139.7 | 143.8 | 133.9 | 140.3 | 140.3 |
| 48 | 140.3 | 144.7 | 135.4 | 141.2 | 141.6 |
| 51 | 140.7 | 145.4 | 136 | 142.1 | 142.9 |
| 54 | 141.5 | 145.9 | 136.8 | 143.2 | 144.4 |
| 57 | 142 | 146.5 | 137.7 | 143.9 | 145.3 |
| 60 | 143.3 | 146.8 | 138.8 | 144.4 | 146 |
| 63 | 144.7 | 147.3 | 139.2 | 144.7 | 146.7 |
| 66 | 146.7 | 148.8 | 141.6 | 145.7 | 148.6 |
| 69 | 147.1 | 149.2 | 142.4 | 145.9 | 148.8 |
| 72 | 148 | 149.2 | 142.9 | 145.7 | 148.9 |

Table S2. Final parameter estimates from fitting the simple Mitscherlich (Eqn. 1) to Dataset 1−25.

| Dataset | Parameter | | |
|---|---|---|---|
| | *c* | *T* | *A* |
| 1 | 0.0138 | 0 | 163.5 |
| 2 | 0.0333 | 0 | 103.1 |
| 3 | 0.0171 | 0 | 124.2 |
| 4 | 0.0187 | 0 | 114.2 |
| 5 | 0.0260 | 0 | 111.79 |
| 6 | 0.0245 | 0 | 132.6 |
| 7 | 0.0350 | 0 | 109.9 |
| 8 | 0.0247 | 0 | 123.7 |
| 9 | 0.0146 | 0 | 133.2 |
| 10 | 0.0136 | 0 | 133.3 |
| 11 | 0.0394 | 0 | 63.9 |
| 12 | 0.0318 | 0.4 | 79.8 |
| 13 | 0.0176 | 0 | 133.8 |
| 14 | 0.0308 | 0 | 103.1 |
| 15 | 0.0270 | 0 | 52.6 |
| 16 | 0.0330 | 0 | 57.4 |
| 17 | 0.0370 | 0 | 58.4 |
| 18 | 0.0710 | 0 | 120.6 |
| 19 | 0.0680 | 0 | 133.3 |
| 20 | 0.0829 | 0.6 | 132.1 |
| 21 | 0.0797 | 0.3 | 144.5 |
| 22 | 0.0781 | 0.5 | 148.7 |
| 23 | 0.0718 | 0 | 140.3 |
| 24 | 0.0927 | 0.6 | 143.9 |
| 25 | 0.0793 | 0.3 | 146.0 |

Table S3. Final parameter estimates from fitting the France model (Eqn. 2) to Dataset 1−25.

| Dataset | Parameter | | | |
|---|---|---|---|---|
| | *c* | *d* | *T* | *A* |
| 1 | 0.0050 | 0.0190 | 0 | 246.8 |
| 2 | 0.0090 | 0.0729 | 0 | 128.6 |
| 3 | 0.0027 | 0.0245 | 0 | 255.8 |
| 4 | - | - | - | - |
| 5 | 0.0048 | 0.0535 | 0 | 166.8 |
| 6 | 0.0053 | 0.0495 | 0 | 193.2 |
| 7 | 0.0079 | 0.0803 | 0 | 139.2 |
| 8 | 0.0080 | 0.0487 | 0 | 159.5 |
| 9 | 0.0050 | 0.0212 | 0 | 201.0 |
| 10 | 0.0082 | 0.0144 | 0 | 160.3 |
| 11 | 0.0159 | 0.0749 | 0 | 72.3 |
| 12 | 0.0256 | 0.0188 | 0 | 82.5 |
| 13 | 0.0093 | 0.0246 | 0 | 161.5 |
| 14 | 0.0193 | 0.0389 | 0 | 111.2 |
| 15 | 0.0270 | 0 | 0 | 52.6 |
| 16 | 0.0076 | 0.0454 | 0 | 90.5 |
| 17 | 0.0041 | 0.0451 | 0 | 117.9 |
| 18 | 0.0506 | 0.0687 | 0.18 | 123.7 |
| 19 | 0.0379 | 0.0956 | 0.13 | 140.2 |
| 20 | 0.0829 | 0* | 0.56 | 132.1 |
| 21 | 0.0797 | 0* | 0.32 | 144.5 |
| 22 | 0.0781 | 0* | 0.47 | 148.7 |
| 23 | 0.0634 | 0.032 | 0.16 | 141.4 |
| 24 | 0.0927 | 0* | 0.56 | 143.9 |
| 25 | 0.0775 | 0.0064 | 0.30 | 146.2 |

* Reverted to simple Mitscherlich (Eqn. 1), $d = 0$

Table S4. Final parameter estimates from fitting the double Mitscherlich model (Eqn. 3) to Dataset 1−25.

| Dataset | Parameter | | | | | |
|---|---|---|---|---|---|---|
| | $A_1$ | $c_1$ | $T_1$ | $A_2$ | $c_2$ | $T_2$ |
| 1 | 60.3 | 0.0928 | 3.1 | 84.3 | 0.0264 | 43.4 |
| 2 | 61.9 | 0.1455 | 2.9 | 62.9 | 0.0169 | 33.3 |
| 3 | 47.6 | 0.1433 | 3.0 | 81.4 | 0.0184 | 35.4 |
| 4 | 47.5 | 0.1923 | 3.0 | 78.6 | 0.0214 | 44.8 |
| 5 | 56.2 | 0.1597 | 2.8 | 66.4 | 0.0224 | 34.3 |
| 6 | 64.6 | 0.1574 | 3.0 | 72.7 | 0.0284 | 35.9 |
| 7 | 67.6 | 0.1479 | 2.7 | 52.3 | 0.0311 | 37.5 |
| 8 | 60.8 | 0.1405 | 2.8 | 66.8 | 0.0273 | 34.6 |
| 9 | 42.8 | 0.1601 | 3.4 | 83.9 | 0.0194 | 32.4 |
| 10 | 43.7 | 0.1143 | 3.6 | 80.7 | 0.0182 | 33.6 |
| 11 | 41.5 | 0.0165 | 3.4 | 40.4 | 0.0142 | 31.6 |
| 12 | 47.0 | 0.1224 | 3.6 | 42.3 | 0.0192 | 29.5 |
| 13 | 48.2 | 0.1524 | 3.1 | 76.4 | 0.0263 | 31.1 |
| 14 | 63.8 | 0.1135 | 3.6 | 75.9 | 0.0107 | 34.4 |
| 15 | 52.6* | 0.0270* | 0 | 0 | 0 | 0 |
| 16 | 57.4* | 0.0332* | 0 | 0 | 0 | 0 |
| 17 | 58.4* | 0.0369* | 0 | 0 | 0 | 0 |
| 18 | 109.7 | 0.0903 | 0.3 | 38.7 | 0.0164 | 37.2 |
| 19 | 112.6 | 0.0986 | 0.2 | 45.0 | 0.0187 | 25.7 |
| 20 | 132.1* | 0.0829* | 0.6* | 0 | 0 | 0 |
| 21 | 142.4 | 0.0832 | 0.4 | 11.2 | 0.0477 | 55.6 |
| 22 | 148.7* | 0.07812* | 0.5* | 0 | 0 | 0 |
| 23 | 138.6 | 0.0744 | 0.1 | 5.0 | 0.4023 | 61.8 |
| 24 | 141.5 | 0.0969 | 0.6 | 6.9 | 0.0383 | 43.0 |
| 25 | 140.4 | 0.0881 | 0.4 | 14.7 | 0.0289 | 38.9 |

* Reverted to simple Mitscherlich (Eqn. 1), $A_2 = 0$.

Table S5. Final parameter estimates from fitting the Mitscherlich + linear (Eqn. 4) to Dataset 1−25.

| Dataset | Model | | | | |
|---|---|---|---|---|---|
| | $A_1$ | $c$ | $T_1$ | $\beta$ | $T_2$ |
| 1 | 63.7 | 0.0776 | 2.8 | 0.9361 | 35.9 |
| 2 | 60.9 | 0.1494 | 2.9 | 0.5779 | 25.1 |
| 3 | 46.7 | 0.1476 | 3.0 | 0.7831 | 27.4 |
| 4 | 47.2 | 0.1959 | 3.0 | 0.8249 | 35.9 |
| 5 | 50.4 | 0.2152 | 3.0 | 0.6922 | 18.1 |
| 6 | 66.7 | 0.1389 | 2.9 | 0.8325 | 29.8 |
| 7 | 67.5 | 0.1452 | 2.6 | 0.6160 | 27.7 |
| 8 | 64.6 | 0.1111 | 2.5 | 0.7229 | 28.7 |
| 9 | 45.2 | 0.1330 | 3.3 | 0.8139 | 28.0 |
| 10 | 50.1 | 0.0771 | 3.1 | 0.7222 | 31.4 |
| 11 | 42.0 | 0.1583 | 3.4 | 0.3400 | 28.1 |
| 12 | 51.5 | 0.0952 | 3.5 | 0.3720 | 27.6 |
| 13 | 71.8 | 0.0475 | 0.6 | 0.6026 | 32.7 |
| 14 | 65.5 | 0.1057 | 3.6 | 0.5385 | 32.8 |
| 15 | 27.5 | 0.0932 | 0.5 | 0.4916 | 29.1 |
| 16 | 35.4 | 0.0856 | 0.5 | 0.4781 | 28.7 |
| 17 | 42.8 | 0.0714 | 0.3 | 0.6583 | 43.7 |
| 18 | 109.6 | 0.0906 | 0.3 | 0.4733 | 34.8 |
| 19 | 114.3 | 0.0954 | 0.1 | 0.5361 | 23.7 |
| 20 | 131.6 | 0.0839 | 0.6 | 0.1346 | 57.5 |
| 21 | 140.5 | 0.0859 | 0.4 | 0.1968 | 36.6 |
| 22 | 147.7 | 0.0794 | 0.5 | 0.0402 | 33.1 |
| 23 | 130.4 | 0.0848 | 0.2 | 0.02497 | 21.1 |
| 24 | 141.5 | 0.0969 | 0.6 | 0.1557 | 40.0 |
| 25 | 140.4 | 0.0879 | 0.4 | 0.2689 | 36.4 |

Figure S1: Observed (●) and predicted gas production profiles resulting from fitting Equations (1) – (4) to Dataset 1 – 7, horses displaying clinical signs of laminitis, from Experiment 1.

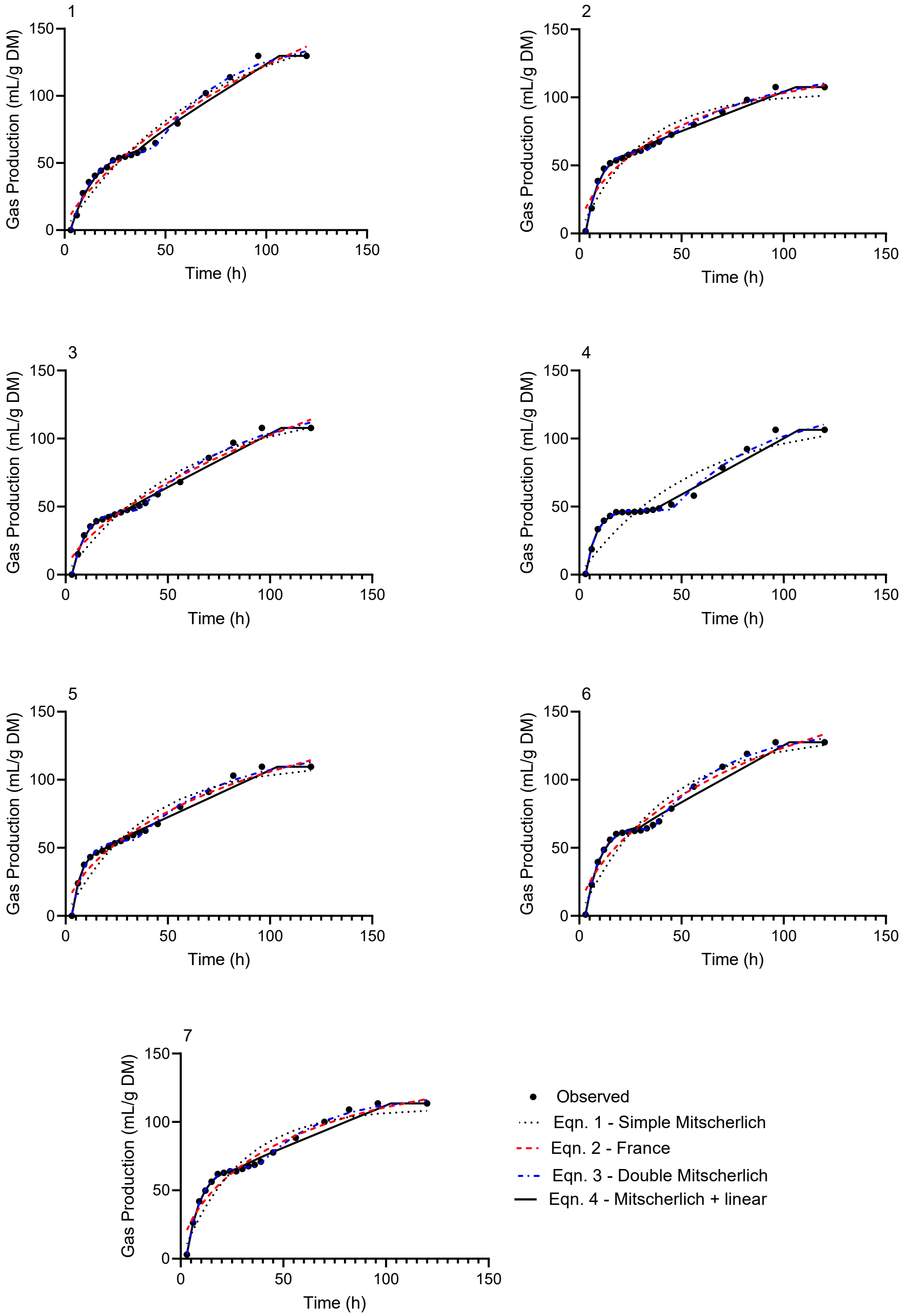

Figure S2. Observed (●) and predicted gas production profiles resulting from fitting Equations (1) – (4) to Datasets 8 – 14, clinically normal horses, from Experiment 1.

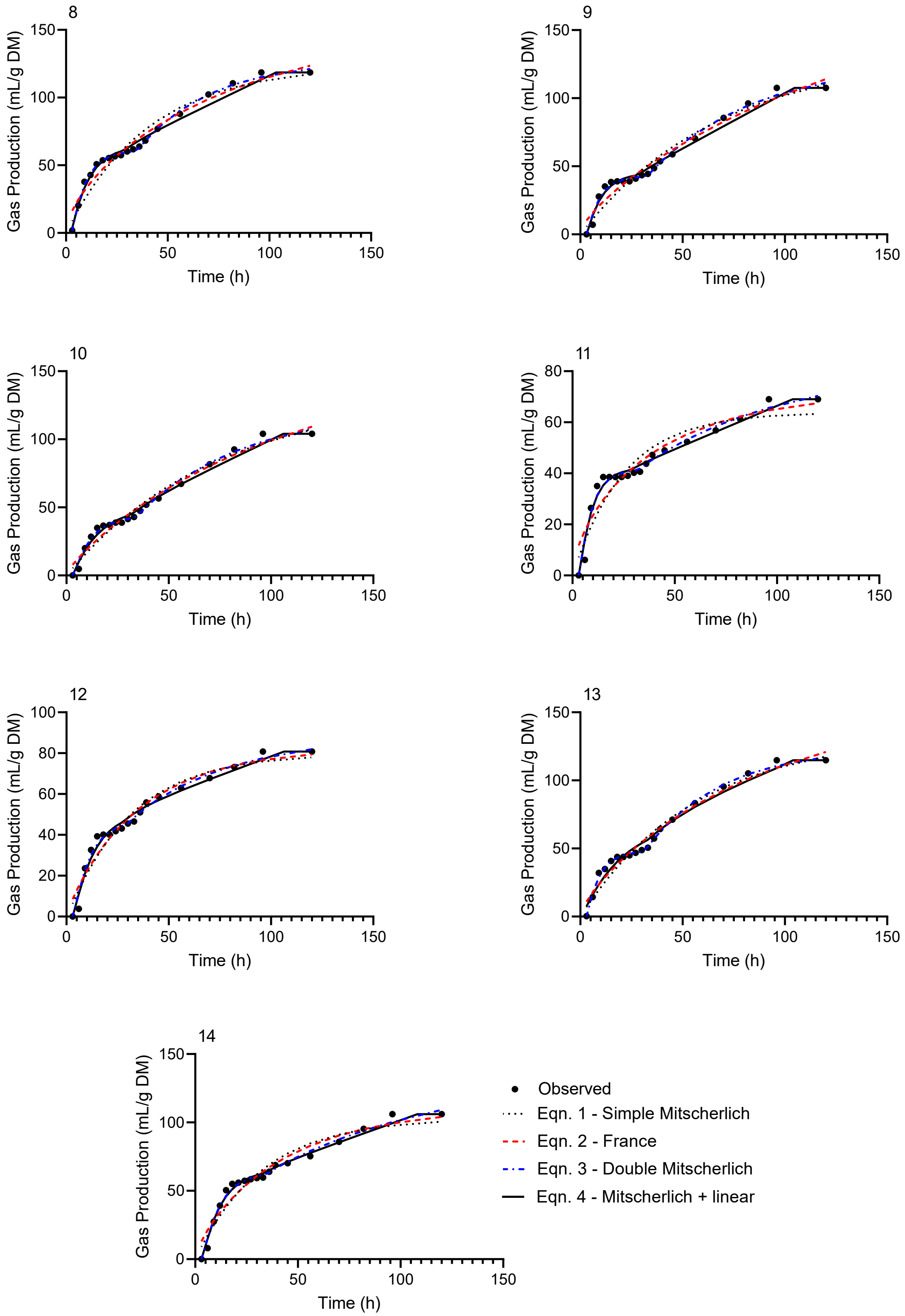

Figure S3. Observed (●) and predicted gas production profiles resulting from fitting Equations (1) – (4) to Dataset 15 – 17, datasets exhibiting atypical dual-phase gas production curves, from Experiment 2.

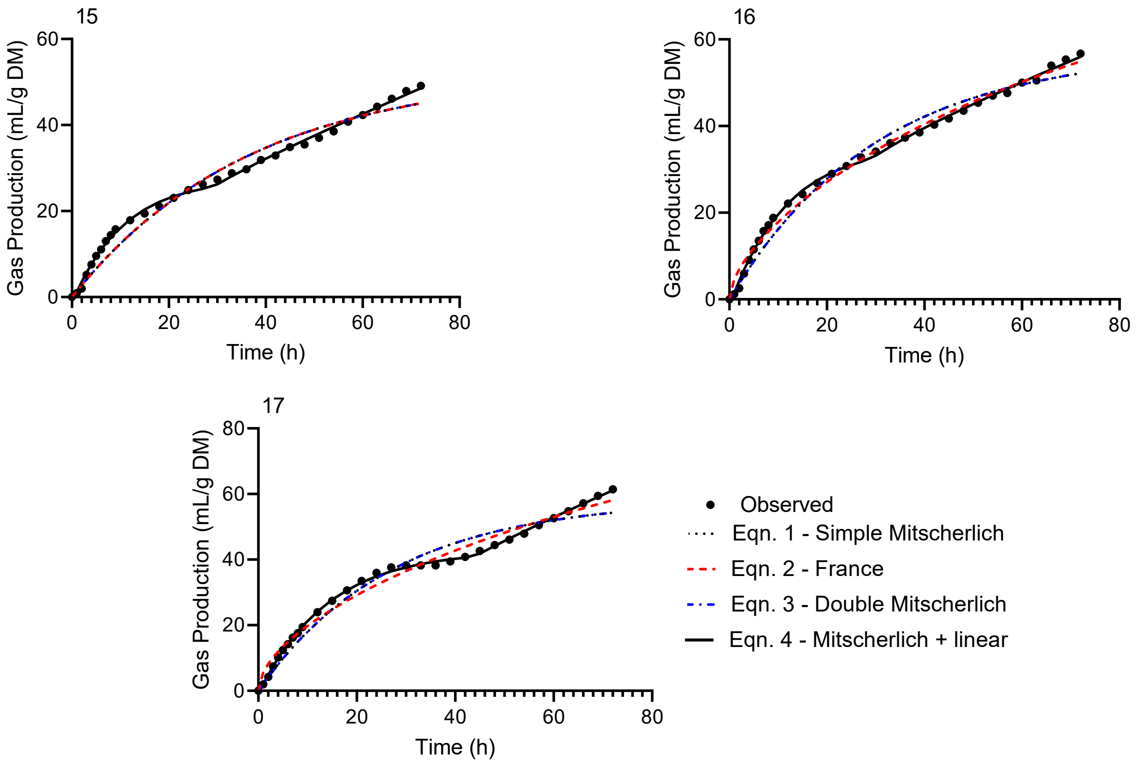


Double Mitscherlich, Equation (3), whereby best fit was achieved with $A_2 = 0$, therefore reverting to a simple Mitscherlich, viz. Equation (1) in Dataset 15–17.

Figure S4. Observed (●) and predicted gas production profiles resulting from fitting Equations (1) – (4) to Dataset 18 – 25, datasets exhibiting typical single-phase gas production curves, from Experiment 2

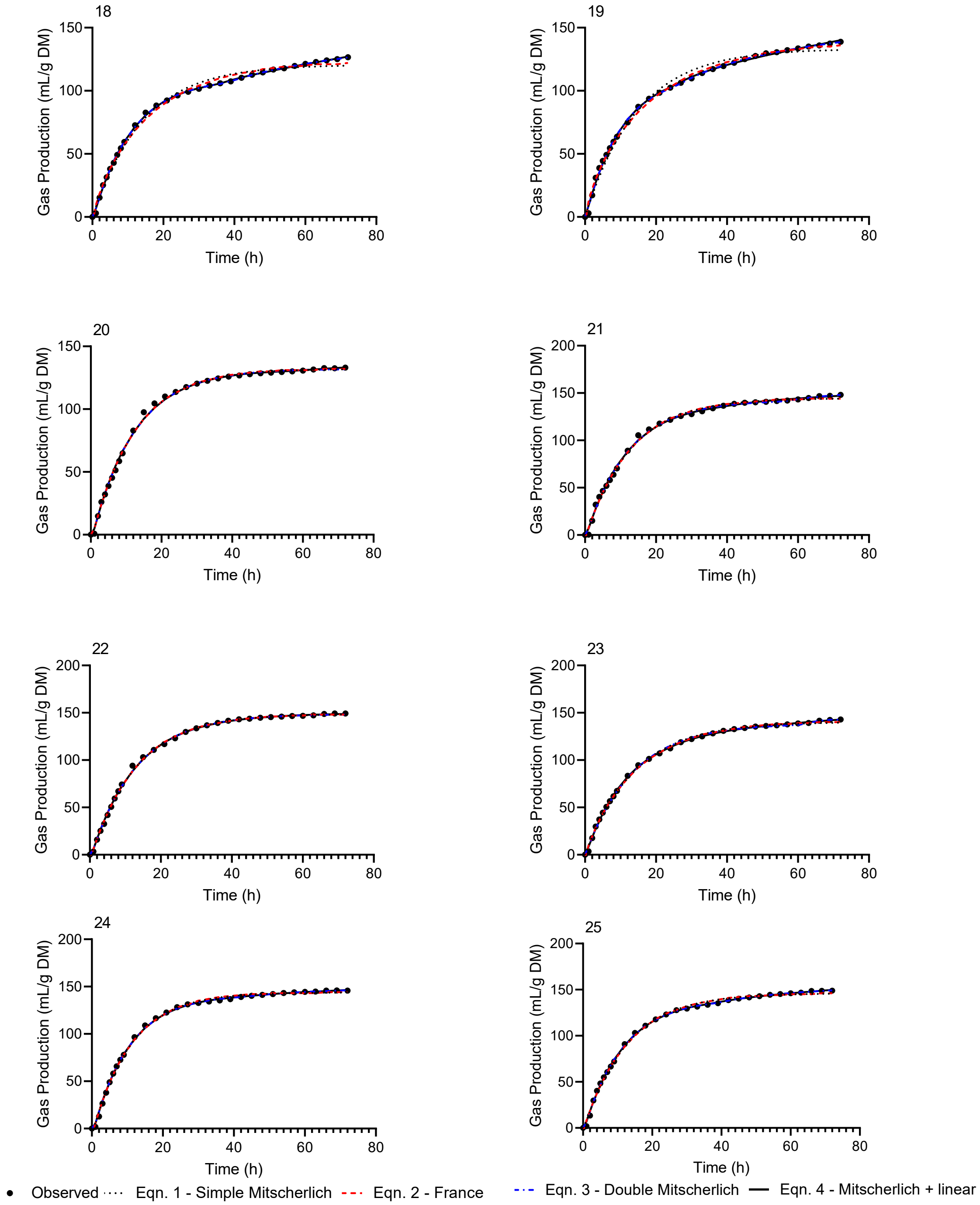


Double Mitscherlich, Equation (3), whereby best fit was achieved with $A_2 = 0$, therefore reverting to a simple Mitscherlich, viz. Equation (1) in Dataset 20 and 22.

France, Equation (2), whereby best fit was achieved when $d = 0$, therefore reverting to a simple Mitscherlich, viz. Equation (1) in Dataset 20–22 and 24.
